# Aggregation-primed molten globule conformers of the p53 core domain provide potential tools for studying p53C aggregation in cancer

**DOI:** 10.1074/jbc.RA118.003285

**Published:** 2018-05-31

**Authors:** Murilo M. Pedrote, Guilherme A. P. de Oliveira, Adriani L. Felix, Michelle F. Mota, Mayra de A. Marques, Iaci N. Soares, Anwar Iqbal, Douglas R. Norberto, Andre M. O. Gomes, Enrico Gratton, Elio A. Cino, Jerson L. Silva

**Affiliations:** From the ‡ Programa de Biologia Estrutural, Instituto de Bioquímica Médica Leopoldo de Meis, Instituto Nacional de Ciência e Tecnologia de Biologia Estrutural e Bioimagem, Centro Nacional de Ressonância Magnética Nuclear Jiri Jonas, Universidade Federal do Rio de Janeiro, Rio de Janeiro, RJ 21941-901, Brazil,; the § Department of Biochemistry and Molecular Genetics, University of Virginia, Charlottesville, Virginia 22908,; the ¶ Laboratory for Fluorescence Dynamics, Biomedical Engineering Department, University of California, Irvine, California 92697-2717, and; the ‖ Departamento de Bioquímica e Imunologia, Instituto de Ciências Biológicas, Universidade Federal de Minas Gerais, Belo Horizonte, MG 31270-901, Brazil

**Keywords:** protein folding, protein aggregation, p53, amyloid, cancer, tumor suppressor, fluorescence spectroscopy, hydrostatic pressure, molten globule

## Abstract

The functionality of the tumor suppressor p53 is altered in more than 50% of human cancers, and many individuals with cancer exhibit amyloid-like buildups of aggregated p53. An understanding of what triggers the pathogenic amyloid conversion of p53 is required for the further development of cancer therapies. Here, perturbation of the p53 core domain (p53C) with subdenaturing concentrations of guanidine hydrochloride and high hydrostatic pressure revealed native-like molten globule (MG) states, a subset of which were highly prone to amyloidogenic aggregation. We found that MG conformers of p53C, probably representing population-weighted averages of multiple states, have different volumetric properties, as determined by pressure perturbation and size-exclusion chromatography. We also found that they bind the fluorescent dye 4,4′-dianilino-1,1′-binaphthyl-5,5′-disulfonic acid (bis-ANS) and have a native-like tertiary structure that occludes the single Trp residue in p53. Fluorescence experiments revealed conformational changes of the single Trp and Tyr residues before p53 unfolding and the presence of MG conformers, some of which were highly prone to aggregation. p53C exhibited marginal unfolding cooperativity, which could be modulated from unfolding to aggregation pathways with chemical or physical forces. We conclude that trapping amyloid precursor states in solution is a promising approach for understanding p53 aggregation in cancer. Our findings support the use of single-Trp fluorescence as a probe for evaluating p53 stability, effects of mutations, and the efficacy of therapeutics designed to stabilize p53.

## Introduction

The role of p53 in different types of cancer arises from the high incidence of monoallelic mutations in TP53 and from the negative dominance effect that mutated p53 imposes on the WT protein, leading to the loss of its tumor suppressor functions or the gain of oncogenic functions (1–4). The participation of WT and mutant amyloid-like aggregates of p53 in different types of cancer is an active topic in cancer research (5–9). In recent years, prevention of p53 aggregation and rescue of its activity when mutated have emerged as possible therapeutic options for treating cancer (10). Examples of the strategies being pursued include peptides that stabilize the folded state (11–13) or occlude its most amyloidogenic region, in β-strand S9 (14). p53 aggregation is also avoided when its native state is stabilized by specific nucleic acid recognition (15, 16).

Therapeutics directed at transient (17) and pre-amyloidogenic molten globule (MG)^5^ p53 conformers may also be effective against some mutant classes (18). MGs are compact forms that preserve secondary structures and exhibit exposure of some nonpolar groups, increased hydrophobic surfaces, rotational isomerization of side-chains, higher loop flexibilities, and volume changes (19–21). The structural equilibrium of many proteins may be perturbed to induce MG states using low concentrations of denaturants or pressure (22, 23). MG forms of some amyloidogenic proteins have been shown to be involved in their structural conversion. For instance, MG states of the human prion protein are precursors to oligomerization (24). Also, MG states of β_2_-microglobulin have been directly linked to its amyloidogenicity (25, 26), and those of mutant human stefin B are as toxic as its aggregates (27). In the case of p53, MG states have been detected in cells under acidic conditions (28), but their involvement in amyloid conversion has not yet been explored.

A major challenge in developing therapies that target these states is their biophysical characterization, which is complicated by their short lifetimes, and tendency to aggregate. The results of molecular dynamic simulations and limited proteolysis assays support the notion that multiple sites in the p53 DNA-binding domain (p53C) may play a role in its aggregation (29, 30). Although the Φ-value analysis was used to map the early transition states of p53 aggregation (31), the structural properties that promote transition into pre-amyloidogenic conformers remain elusive.

The characterization of pre-aggregate MG conformations of p53 could provide insights for novel cancer therapeutics. Although the short-lived conformers of p53 are challenging to assess inside cells, the combination of recombinant p53, fluorescence spectroscopy, and phasor plots serves as a powerful tool for studying these species *in vitro*. Using these methodologies, together with chemical (guanidine hydrochloride (GuHCl)) and physical (hydrostatic pressure) approaches, we explored the energetic landscape of p53C and its homologs p63C and p73C and elucidated features specific to its pre-amyloidogenic states. In addition, a methodology for trapping particular conformers in solution is demonstrated. The findings are expected to permit the characterization of p53 mutational variants and screening of potential stabilizing molecules.

## Results

### Stability of p53 family members against GuHCl

**Figure 1.**
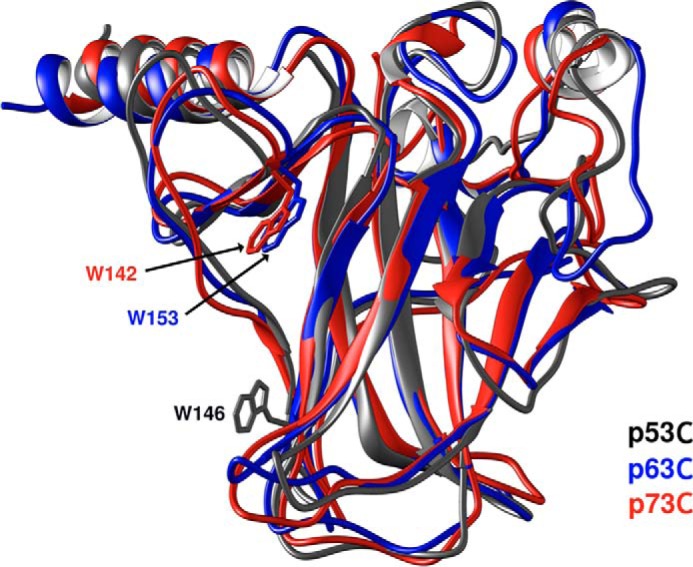
**Superimposed crystal structures of the DNA-binding domains of p53, p63, and p73 (Protein Data Bank codes 2FEJ (51), 2RMN (52), and 2XWC (53), respectively), highlighting the positions of the single Trp residues.**

**Figure 2.**
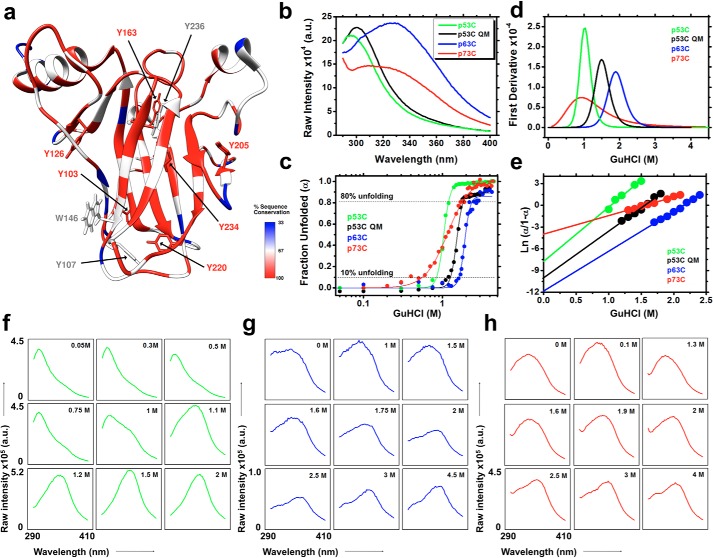
*a*, percentage of sequence conservation among the p53 family members (p53C, p63C, and p73C) is depicted as a *color code* in the p53 core domain structure (Protein Data Bank code 2FEJ). Tyr residues and the single Trp are shown as *sticks. b* and *c*, fluorescence emission spectra recorded upon excitation at 280 nm and the fraction unfolded plotted against GuHCl for the p53C family members. *d*, first derivative plots used to extract the GuHCl concentration at the midpoint of the transitions (*G*_50%_). *e*, Δ*G*_0 M_^F-U^ values were obtained from the intercepts of the linear regression of the transition points multiplied by the temperature and the universal gas constant. *f–h*, raw fluorescence spectra at different GuHCl concentrations for p53C (*f*), p63C (*g*), and p73C (*h*). GuHCl concentrations were chosen according to their fraction unfolded plots (*c*) to better represent the spectroscopic events discussed in this work. The *scales* in the *bottom left graphs* are the same for each construct, unless otherwise stated.

Unlike the Trp in p63C and p73C, which localizes to the beginning of β-strand S2, Trp-146 in p53C is located at the end of β-strand S3 (Fig. 1). This local probe position, together with eight Tyr residues, results in a different emission spectrum compared with its homologs, probably due to quenching of the Trp signal (around 350 nm) by the surrounding Tyr rings in the native state, which is progressively lessened during unfolding (Fig. 2, *a* and *b*).

First, the stabilities of p53C, p53C QM (a designed higher-stability mutant) (32), p63C, and p73C in GuHCl were investigated (Fig. 2*c*). Successive experiments at different GuHCl concentrations were performed to obtain data points covering the pretransition and transition states (Fig. 2*c*). Subdenaturing concentrations of GuHCl, corresponding to <10% unfolding, ranged from 0.01 (∼2% unfolding) to 0.8 m (7.5% unfolding) for p53C and from 0.01 to 1.2 m GuHCl (10% unfolding) for p53C QM (Fig. 2*c*). The GuHCl concentrations at the midpoints of the transitions were obtained from the first derivatives of the fits (Fig. 2*d* and Table 1). Also, the free energies of denaturation were ascertained by extrapolation of the linear regression intercepts of the transition points multiplied by the temperature and the universal gas constant (Fig. 2*e* and Table 1; see Equation 5). The free energies of denaturation revealed the order of stabilities: p63C > p53C QM > p53C > p73C. Below, the physical and aggregation properties of p53C species under these subdenaturing conditions and in combination with HHP are investigated.

**Table 1:** **Thermodynamic parameters**

	p53C	p53C QM	p63C	p73C
*G*_50%_ (m)	1.03	1.49	1.88	0.92
Δ*G*_0 m_^F ↔ U^ (kcal/mol)	4.6 ± 0.46	5.95 ± 0.29	7 ± 0.22	2.4 ± 0.08

### Phasor plots reveal hidden states of p53 family members

**Figure 3.**
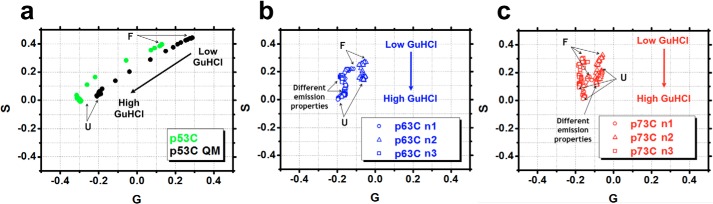
**Phasor plots for the GuHCl data sets for the p53C family members in independent preparations (*n1–n3*) (*a–c*).** The robustness of the p53C data set is not shown due to the curves being highly reproducible. *G* and *S* represent the first harmonic Fourier transformation of the spectral axis wavelength and intensity, respectively. Collection of folded and unfolded ensembles are indicated as *F* and *U*, respectively.

The phasor representation is obtained by the first harmonic Fourier transformation of the wavelength and intensity values of a series of emission spectra (Fig. 2, *f–h*; Equations 8 and 9) and is a robust strategy to reveal additional states within a two-state model (33, 34). Plotting the emission spectra at all studied GuHCl concentrations as phasors revealed different behavior among the p53 family members (Fig. 3, *a–c*). The line of linear combination refers to two species (*i.e.* F → U). If the species are changing in abundance, the corresponding phasor will move along the linear combination line, as observed in Fig. 3*a*, suggesting a two-state model for p53C and p53C QM during GuHCl-induced unfolding. In contrast, a deviation from the single straight line will occur if other species with different fluorescence emission properties are populated. This behavior seems to be the case for the deviation from linearity in the unfolding of p63C and p73C (Fig. 3, *b* and *c*).

### Evidence of p53 conformations with MG features

**Figure 4.**
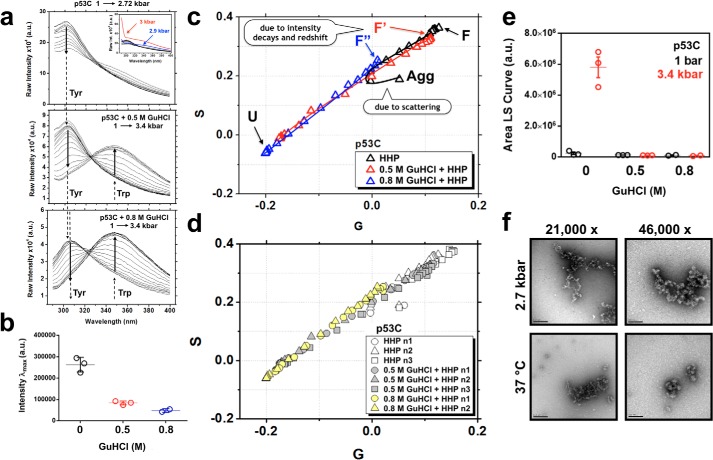
*a*, fluorescence emission data sets for p53C upon excitation at 280 nm showing HHP titrations in 0 m (*top*), 0.5 m (*middle*), and 0.8 m (*bottom*) GuHCl. The Tyr and Trp contributions are *labeled*. Note the slight red shift in the Tyr signal upon incubation with GuHCl at the above concentrations. The *inset* shows in *blue* and *red* the scattering contribution at pressures higher than 2.7 kbar. *b*, *scatter plot* of the maximum peak intensities of p53C in the absence (*black*) and presence of 0.5 (*red*) and 0.8 m GuHCl (*blue*) at atmospheric pressure. *c*, phasor plots of the data sets described in *a*, highlighting the population-weighted average states of folded (*F*), folded containing MG-like properties (*F*′ and *F*″), and unfolded (*U*) at increasing GuHCl concentrations. Pressures higher than 2.7 kbar shift the p53C energetic landscape to a dead-end aggregation route (*Agg*), which is characterized by an inflection in the phasor plot. *d*, different data sets were obtained in the absence or in the presence of 0.5 or 0.8 m GuHCl for p53C to confirm the robustness of the measurements. *e*, *scatter plots* of the area under the LS curve at 1 and 3.4 kbar for the data sets described in *a. f*, negatively stained micrographs showing the morphology of the dead-end aggregation route of p53C after incubation at 2.7 kbar for 2 h or at 37 °C. *a.u.*, arbitrary units.

Because GuHCl-induced unfolding of p53C revealed a two-state process (Fig. 3*a*), subdenaturing concentrations of GuHCl and HHP were combined to explore better the properties of p53C along the F → U transition. The emission spectra of p53C at atmospheric pressure in the absence of (Fig. 4*a*, *top*) or immediately after the addition of 0.5 m (Fig. 4*a*, *middle*) and 0.8 m GuHCl (Fig. 4*a*, *bottom*) revealed a consistent intensity decrease from ∼260,000 arbitrary units in the absence of GuHCl to ∼85,000 at 0.5 m and ∼45,000 at 0.8 m. This is better observed in the scatter plots of the maximum peak intensities (Fig. 4*b*). Furthermore, a slight but consistent red shift from λ_max_ = 302 nm at 0.5 m to λ_max_ = 306 nm at 0.8 m GuHCl was observed (Fig. 4*a*, *vertical dashed lines*). The intensity decays accompanied by the red shifts upon subdenaturing GuHCl increase at atmospheric pressure, which suggests a slight exposure of Tyr residues.

HHP titration in the absence of GuHCl revealed that pressures >2.7 kbar lead to an abrupt increase of the intensity counts close to the region of the excitation peak as a result of scattering events (Fig. 4*a*, *top*, *inset*). This behavior was not observed when 0.5 or 0.8 m GuHCl was added immediately before the HHP increment series (Fig. 4*a*, *middle* and *bottom*). The result was suggestive of pressure-induced aggregation of p53C in the absence of GuHCl, which was completely abolished when subdenaturing amounts of GuHCl were added immediately before HHP application. Phasor analysis (Fig. 4, *c* and *d*) of the emission spectra and light scattering measurements (Fig. 4*e*) confirm the HHP-induced aggregation of p53C and its inhibition when combined with low GuHCl concentrations. Negative staining also revealed the aggregation of HHP-perturbed p53C, as similar oligomeric species to those induced by temperature were observed (Fig. 4*f*).

The inflection in the phasor trajectory of the HHP titration without GuHCl arose from scattering at pressures >2.7 kbar and indicated a shift from unfolding to aggregation (Fig. 4*c*, *Agg*). The offset positions, F′ and F″, from the initial folded F state (Fig. 4*c*) arose from changes in intensity counts and red shifts when subdenaturing GuHCl was progressively increased from 0 m to 0.5 and 0.8 m. F′ (0.5 m GuHCl before HHP) localized very close to F on the phasor plot and corresponded to a highly native-like population. The F″ species, representing the 0.8 m GuHCl condition before application of HHP, fell near the aggregation inflection point, indicative of an ensemble of conformers with altered properties compared with F and F′, which are detailed below.

It is important to stress that at 0.5–0.8 m GuHCl, in the absence of HHP, the results do not imply the formation of individual species. Instead, the F′ and F″ “states” probably represent population-weighted averages of multiple conformers that are trapped in solution during the two-state unfolding of p53C. These trapped conformers present, on average, slightly higher exposure of Tyr residues compared with unperturbed p53C (Fig. 4, *a–d*). For p63C and p73C, the phasor deviation from a straight line in Fig. 3 (*b* and *c*) mostly represents changes in the fluorescence emission properties of the excited Trp probe (Fig. 2, *b*, *g*, and *h*) and reflects the presence of multiple conformers during GuHCl-induced unfolding.

**Figure 5.**
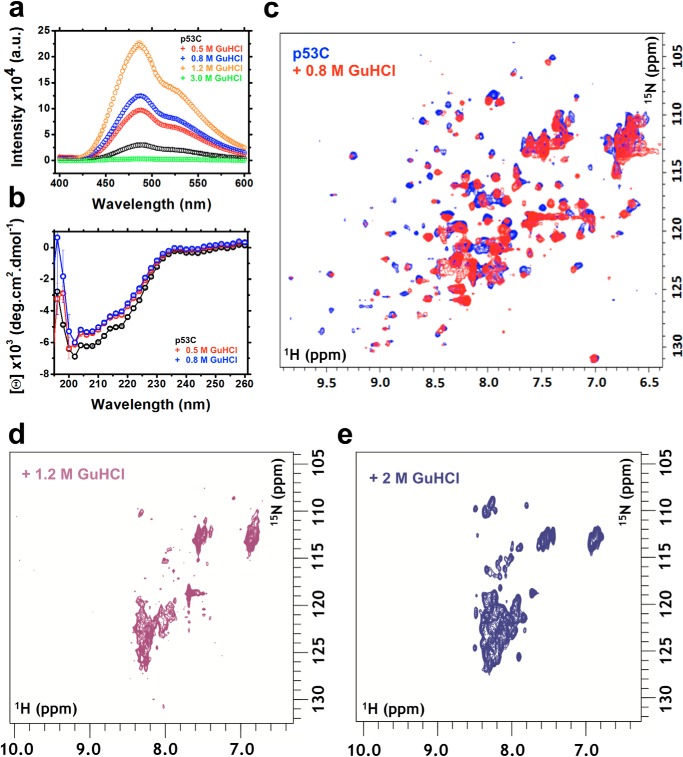
*a*, fluorescence emission spectra of bis-ANS binding to p53C at different GuHCl concentrations. *b*, CD measurements of p53C in the absence or presence of 0.5 or 0.8 m GuHCl. *c–e*, ^1^H-^15^N HSQC spectra of p53C in the absence and presence of 0.8 m (*c*), 1.2 m (*d*), and 2 m (*e*) GuHCl. *a.u.*, arbitrary units.

Binding of bis-ANS (Fig. 5*a*) showed that F′ and F″ had increased exposure of hydrophobic segments but similar secondary structure (Fig. 5*b*) as p53C in the absence of GuHCl, which are hallmarks of MG states. The binding of bis-ANS was ∼1.3- and 1.5-fold higher for the F′ and F″ states, respectively, compared with in the absence of GuHCl. At 1.2 m GuHCl, a condition in which ∼79.5% of the p53C was denatured, as determined by intrinsic fluorescence (Fig. 2*c*), the binding of bis-ANS was ∼3-fold higher relative to 0 m GuHCl, revealing further exposure of hydrophobic segments but the persistence of some residual structure. At 3 m GuHCl, bis-ANS fluorescence was abolished entirely, implying complete unfolding of p53C (Fig. 5*a*).

^1^H-^15^N HSQC experiments in the presence of 0.8 m GuHCl revealed similar ^1^H dispersion as in the absence of GuHCl (Fig. 5*c*). The result suggests that there are few changes in tertiary structure within the ensemble, providing additional evidence of a population of native-like MG states of p53C in 0.8 m GuHCl. At higher GuHCl concentrations (1.2 and 2 m), the NMR spectra revealed unfolding (Fig. 5, *d* and *e*) in agreement with fluorescence data (Fig. 2*c*).

Together, the data show the modulation of the p53C population-weighted average states by GuHCl and HHP perturbation. The properties, mapped by phasor interpretation, bis-ANS, CD, fluorescence spectroscopy, and NMR, are suggestive of the presence of MG conformers within the p53C ensemble.

### Volumetric properties of p53 states

To further dissect the physical properties of the F′ and F″ MG states, the volumetric properties of p53C under HHP and in the absence and presence of 0.5 or 0.8 m GuHCl were investigated.

Pressure alters the volumetric properties of biomolecules and can be applied to study protein folding landscapes (35). Here, volume changes (Δ*V*) refer to the expansion or retraction of the ensemble of states along the F ↔ U transition, as measured from HHP titrations. Because in the absence or presence of 0.5 and 0.8 m GuHCl all HHP curves reached the unfolded state, the volume differences (Δ*V*) obtained from these three GuHCl conditions (0, 0.5, and 0.8 m) directly report the volume deviations of p53C between the different conditions.

**Figure 6.**
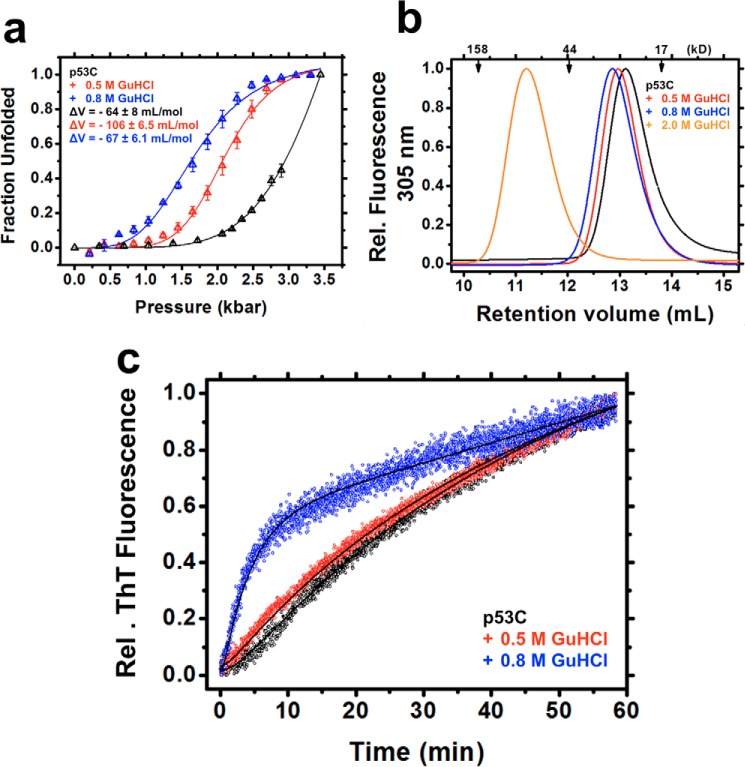
*a*, fraction unfolded plotted against pressure based on titration in the absence or presence of 0.5 or 0.8 m GuHCl. For the Δ*V* calculations, see “Experimental procedures.” *b*, size-exclusion chromatography recorded at a fluorescence emission of 305 nm upon excitation at 280 nm. *c*, thioflavin T kinetics obtained for the competent pre-amyloidogenic states within the population-weighted average of p53C states in the absence and presence of 0.5 or 0.8 m GuHCl at 37 °C. The curve fits (see Equation 42) are represented as *black lines*.

The volume changes of F ↔ U (*i.e.* 0 m GuHCl), F′ ↔ U (*i.e.* 0.5 m GuHCl), and F″ ↔ U (*i.e.* 0.8 m GuHCl) ensembles were obtained from the transition points of the HHP titrations (see “Experimental procedures”) after plotting the raw fluorescence data (Fig. 4*a*) as unfolded fractions against the pressure values (Fig. 6*a* and Table 2). The findings suggest an expanded set of conformers at F′ (Δ*V*^F′ ↔ U^ = −106 ± 6.5 ml/mol), followed by volume retraction at F″, which was similar to the native state Δ*V*^F ↔ U^ = −64 ± 8 and Δ*V*^F″ ↔ U^ = −67 ± 6.1 ml/mol. Retention volumes from analytic size-exclusion chromatography also reflected the volume differences (hydrodynamic volumes), with F″ eluting first (∼12.81 ml), followed by F′ (∼12.98 ml) and F (∼13.11 ml) (Fig. 6*b*). These species represent expanded, but still folded, MG-like forms in equilibrium with native and unfolded states. At 2 m GuHCl, retention volume was decreased considerably (∼11.2 ml), indicative of expanded unfolded species (Fig. 6*b*). The different Δ*V* values at a subdenaturing concentration of GuHCl provide additional evidence for the existence of various MG states within the folded ensemble of p53C.

**Table 2:** **Volumetric properties**

	p53C			p53C QM	
	0 m GuHCl	0.5 m GuHCl	0.8 m GuHCl	0 m GuHCl	1.2 m GuHCl
Δ*V* (ml/mol)*^a^*	64.4 ± 8.1*^b^*	105.9 ± 6.5	67.2 ± 6.1	61.2 ± 5.3*^b^*	60.5 ± 3.9

*^a^* Volume changes are expressed as the average ± S.E. (*n* = 3 independent protein preparations).

*^b^* Obtained through extrapolation of the data to 100% of unfolding.

### Aggregation-primed conformers of p53C

Consistent with observations of other amyloidogenic proteins (24–27), MG states of p53C may represent amyloid precursors. A higher aggregation rate for the F″ states, but not for the F′, was observed by thioflavin T (ThT) kinetics (Fig. 6*c*). Compared with 0 and 0.5 m GuHCl, the 0.8 m condition led to rapid aggregation of p53C without a lag phase, suggesting an increased population of aggregation-primed species in solution. The absence of a lag phase and higher rate constants for F′ (Table 3) revealed the existence of aggregation-competent conformers, possibly with decreased folding cooperativity, within the ensemble, which rapidly aggregated at physiological temperature (27). The rate constants *k*_1_ and *k*_2_ are sometimes used interchangeably, with a numerically higher rate constant, *k*_1_, succeeding *k*_2_, which is also why the kinetic analysis data are reported in apparent terms. As such, it was not possible to attribute *k*_1_ or *k*_2_ to the F → I or I → U conversion step of the kinetic model.

**Table 3:** **Kinetic parameters**

	p53C		
	0 m GuHCl	0.5 m GuHCl	0.8 m GuHCl
*k*_1_ (min^−1^)*^a,b^*	0.025 ± 0.21 × 10^−3^	0.033 ± 0.22 × 10^−3^	0.25 ± 0.0046
*k*_2_ (min^−1^)*^a,b^*	0.35 ± 0.016	0.72 ± 0.065	2.22 ± 0.12
*R*^2^	0.9989	0.9993	0.9989

*^a^* Mean and S.E. for all measurements at 5 μm protein.

*^b^ k*_3_ and *a* values were close to zero.

### MG properties persist regardless of increased p53C stability

**Figure 7.**
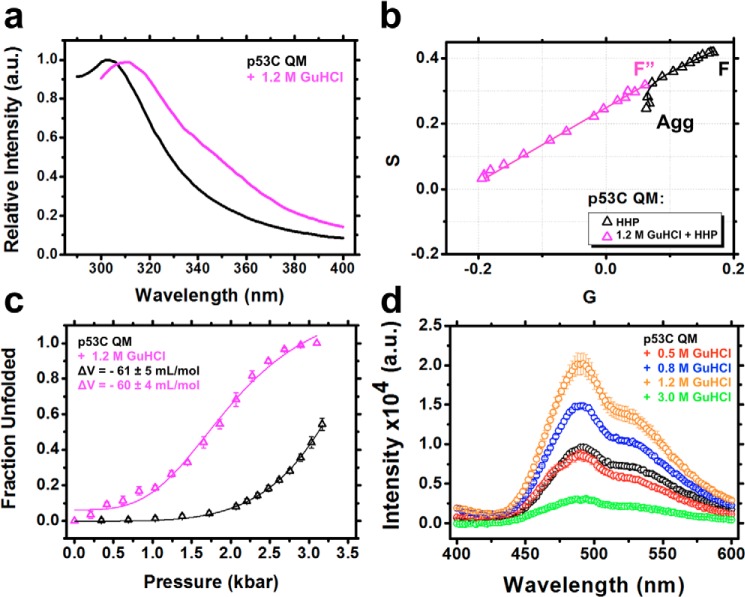
*a*, normalized fluorescence emission spectrum of p53C QM in the absence or in the presence of 1.2 m GuHCl. *b*, phasor plot of p53C QM from the hydrostatic pressure titration data sets in the absence or in the presence of 1.2 m GuHCl. *F*″, the population-weighted average states containing competent MG pre-amyloidogenic conformers. Even p53C QM shifts to a dead-end aggregation route (*Agg*) at pressures higher than 3.2 kbar. *c*, fraction unfolded against pressure titration in the absence or presence of 1.2 m GuHCl. *d*, fluorescence emission spectra of bis-ANS bound to p53C QM in the absence or presence of different GuHCl concentrations. *a.u.*, arbitrary units.

Similarly to p53C, in the presence of subdenaturing GuHCl (<10% unfolding), the superstable p53C QM construct exhibited a slight red shift of the Tyr signal, with no evidence of Trp exposure (Fig. 7*a*). This condition abolished pressure-induced aggregation, as revealed by the linear trajectory on the phasor plots (Fig. 7*b*). The Δ*V* values were similar to the situation without GuHCl (Fig. 7*c* and Table 2), but a 2-fold increase in bis-ANS binding was observed in the presence of 1.2 m GuHCl (Fig. 7*d*). Therefore, despite the higher stability of p53C QM compared with p53C, it adopts MG states with similar properties under mild GuHCl perturbation. In fact, when compared with p53C (Fig. 4*c*) the data for p53C QM in the presence of GuHCl (F″) seem to fall more precisely on the line in the absence of GuHCl before the latter departs from linearity. Although the small differences are within the error of the measurements, the best fit of the F″ conformer in the case of p53C QM may be due to its higher stability when compared with p53C.

### p53C MG states partially protect the single Trp

**Figure 8.**
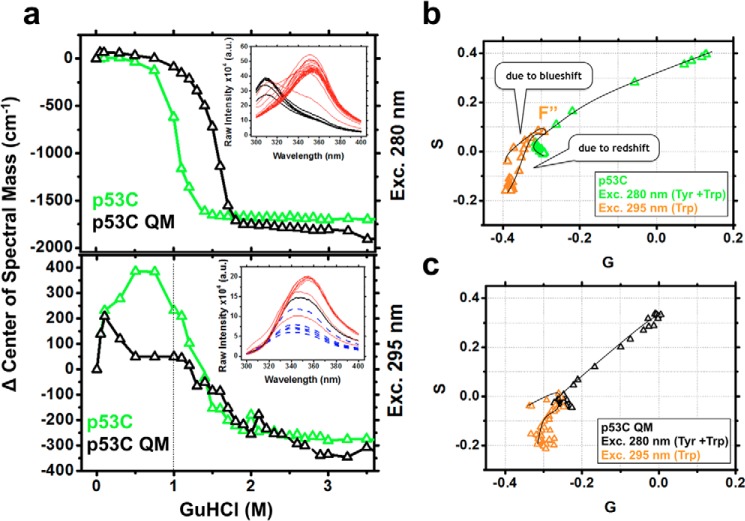
*a*, changes in the center of spectral mass as a function of the GuHCl concentration showing the contribution of Tyr + Trp (*top*) and Trp (*bottom*) for p53C and p53C QM. Note an increase at 400 cm^−1^ in the range of 0–1 m GuHCl for p53C. *Insets*, p53C raw emission data. *Black* and *red curves*, pre- and post-transition spectra. *Blue dashed lines*, blue shift of the Trp in the reported range before exposure to the solvent. *b* and *c*, phasor plots for p53C and p53C QM, respectively, highlighting the conformational changes of the single Trp (*F*″) within the population-weighted average states, as observed from the deviation in the linearity of the plots. *G* and *S*, first harmonic Fourier transformation of the spectral axis wavelength and intensity, respectively.

Filtering the Tyr signal from the emission spectra of p53C (Fig. 8*a*, *top*) and using excitation at 295 nm captured a clearer picture of the unique Trp transition for p53C and p53C QM at subdenaturing GuHCl concentrations. In the absence of GuHCl, the single Trp of p53C was already partially exposed compared with other single Trp proteins (36). Positive changes in the center of spectral mass were detected in the GuHCl range of the F′ and F″ states (Fig. 8*a*, *bottom*; ∼400 cm^−1^). This positive change can be explained by a concomitant intensity decrease and blue shift of the p53C spectra (Fig. 8*a*, *bottom inset*, *blue dashed lines*), which may reflect fluorescence quenching of the Trp signal, possibly due to partial shielding from the solvent by nearby side chains. Structurally, it means that p53C Trp is more exposed than in the presence of subdenaturing concentrations of GuHCl (F′ and F″ states). For p53C QM, the positive change was half of that observed for p53C (Fig. 8*a*, *bottom*; ∼200 cm^−1^) and occurred at lower GuHCl concentrations (<0.3 m). At concentrations of >1 m GuHCl, the single Trp showed progressive red shifts that culminated in full probe exposure (*inset* of Fig. 8*a*, *bottom*, *red lines*). The absolute values of the spectral center of mass in cm^−1^ for p53C were 28,425 in the absence of GuHCl, 28,811 at 0.5 m, 28,809 at 0.8 m, 28,657 at 1 m, and 28,130 at 4.5 m, representing total Trp exposure.

The phasor line of the Trp probe along the GuHCl titration in which the Tyr signal was suppressed exhibited deviations in linearity because of the population of conformers with less exposed Trp (blue shift) at intermediate GuHCl concentrations (Fig. 8, *b* and *c*, *orange triangles*). Thus, p53C Trp becomes less presented initially (F′ and F″ states). At higher GuHCl concentrations, the Trp becomes progressively exposed to the solvent.

## Discussion

In this work, we elucidated features of p53C pre-amyloidogenic states by perturbation of its folding equilibrium using different combinations of subdenaturing concentrations of GuHCl and HHP. Fluorescence experiments revealed conformational changes of the single Trp and Tyr residues before unfolding and the presence of MG conformers, some of which were highly aggregation-prone.

The mechanisms by which chemical denaturants (*e.g.* GuHCl and urea) perturb the native ensemble of proteins have still not been fully clarified. The main theories suggest that the agents may bind directly to protein surfaces or act indirectly by changing the structure of water molecules around proteins, culminating in hydrophobic solvation (37, 38). In the case of HHP, pressure forces water molecules into the protein, favoring disassembly of nonexposed cavities (39) and increased hydration of hydrophobic groups, leading to decreased partial molar volumes (35). The combination of chemical denaturants and HHP intensifies the destabilizing effects, as described by our “push-and-pull” model (40) and further explored by MD simulations (41). When subdenaturing GuHCl concentrations were combined with HHP (Figs. 4*a* (*middle*, *bottom*) and 6*a*), p53C became more susceptible to pressure-induced unfolding, as evaluated by the pressure midpoint values (*p*_½_ = 2.9 kbar in the absence of GuHCl, ∼2 kbar at 0.5 m, and ∼1.6 kbar at 0.8 m). These mild GuHCl concentrations progressively enriched destabilized conformers within the population-weighted ensemble (Fig. 6*a*). When applied separately, GuHCl (Figs. 2*c* and 3*a*) and HHP (Fig. 4*a*, *top*) revealed a modulation of the energetic landscape of p53C from unfolding (GuHCl titration) to aggregation (HHP titration). It is possible that GuHCl binding to p53C prevented nonspecific intermolecular interactions between p53C molecules, leading to unfolding. In contrast, continuous hydration of hydrophobic groups upon HHP application probably favored hydrophobic collapse and aggregation of p53C at pressures >2.7 kbar (Fig. 4, *e* and *f*).

Several amyloidogenic proteins have been shown to adopt conformers with MG features, indicating their relevance as amyloid precursors (24–27). The MG species of p53C displayed partial Tyr exposure (Figs. 4*a* and 7*a*), different Trp fluorescence emission properties (Fig. 8, *a–c*), increased hydrophobic exposure (Figs. 5*a* and 7*d*), persistence of native-like secondary (Fig. 5*b*) and tertiary (Fig. 5*c*) structure, and different volumes compared with the native state (Figs. 6*a* and 7*c*).

Recent evidence has expanded the definition of MGs to dry and wet MG states, suggesting that a collection of related conformers can coexist (42–47). Dry MG states occur as a result of a “hydrogen bond shift” due to direct chemical binding and pulling of water molecules away from the protein solvation shell (40). Although this ensemble of states is challenging to investigate, the use of mild denaturant concentrations and HHP has proven useful to characterize dry MG states of different proteins (40). For p53C, at 0.5 m GuHCl, it was possible to gently disturb the population-weighted average states, revealing an expanded volume change for the ensemble (Fig. 6*a*), while maintaining the essential interactions necessary for tertiary architecture (Fig. 4, *a–c*). During the exchange of solvent and denaturant at the protein surface, some interactions, such as backbone hydrogen bonds (BHBs), could be loosened, resulting in swollen polypeptide conformations. The disruption of some protein-solvent interactions due to GuHCl binding and water expulsion from protein surroundings probably explains the volume expansion of p53C conformers under this condition. The existence of dry MG-like conformers is supported by prior observations showing increased per-residue Δ*V* values, as measured from sigmoidal transitions in high-pressure NMR experiments, and global Δ*V* measurements from fluorescence spectroscopy (40).

A small increase in the concentration of GuHCl from 0.5 to 0.8 m led to volume retraction and slightly higher hydrophobic exposure of the overall population-weighted average of states. The collection of states at 0.8 m may represent the rate-limiting step for both routes (*i.e.* unfolding or aggregation). As shown by ThT kinetics, the ensemble of conformers at 0.8 m GuHCl is highly prone to amyloidogenic aggregation at physiological temperature (Fig. 6*c*). Under this condition, the subtle modulation of interactions (*i.e.* slight exposure of Tyr residues and partial Trp occlusion) is sufficient to alter intra- and intermolecular p53C interactions, culminating in a substantial population of aggregation-primed species.

When higher GuHCl concentrations were used (*i.e.* 1.2 or 2 m), the ensemble of conformers deviated toward increasingly unfolded forms with wet MG properties, as supported by the loss of ^1^H NMR dispersion (Fig. 5, *d* and *e*), higher bis-ANS fluorescence (Fig. 5*a*), Trp exposure (Fig. 2*f*), and lack of aggregation in the HHP titration series (Fig. 4, *c* and *e*). These wet MG species at GuHCl >1 m probably represent the combined effects of GuHCl-induced destabilization of protein-solvent interactions and water infiltration of the hydrophobic core, disrupting the overall fold while maintaining some secondary structure. The presence of poorly protected BHBs seen in MD simulations of p53C could facilitate GuHCl binding, causing structure destabilization and water infiltration (29).

In contrast to p53C, p63C and p73C exhibited different unfolding and aggregation properties (Figs. 3 (*a–c*) and 8 (*b* and *c*)). p73C revealed decreased unfolding cooperativity when compared with p53C, QM, and p63C, as evidenced by a more gradual slope of its GuHCl curve (Fig. 2*c*). Further, the aggregation tendencies of p63C and p73C are negligible compared with p53C when assessed by HHP (29), which may be due to improved protection of BHBs in p63C and p73C, leading to better resistance against water infiltration. The presence of higher exposure of BHBs of p53C is in line with the presence of more transient or unlocked interactions, a typical feature of MG states (44). The cooperative and aggregation dissimilarities observed for the p53C family members are intriguing, considering the ∼60% identity, and are the topic of ongoing studies.

**Figure 9.**
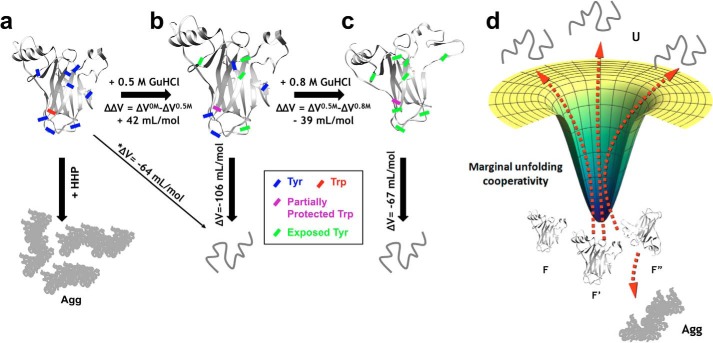
**Combining subdenaturing concentrations of GuHCl with HHP allowed the characterization of population-weighted average states of p53C.** The ensemble of native states (*a*) is converted to a population of states containing expanded volumes (Δ*V* = −106 ml/mol) with intermediate levels of hydrophobic exposure (*b*). Slightly increasing GuHCl leads to a volume retraction (Δ*V* = −67 ml/mol) and additional hydrophobic exposure (*c*). In these states, the local Trp probe is partially protected before full exposure. The use of pressures higher than 2.7 kbar leads p53C to an irreversible dead-end aggregation route (*Agg*). *, obtained through extrapolation to the data values at 100% unfolding. *d*, representation of the marginal unfolding cooperativity of the p53C ensemble of conformers upon small additions of GuHCl.

Although p53C does not behave like a one-state downhill folder (*i.e.* minimal free energy barriers during unfolding), it possesses marginal unfolding cooperativity (48–50), which permits tuning from unfolding to aggregation landscapes, depending on whether chemical or physical forces are applied (Fig. 9, *a–d*). Notably, the conditions at 0.5 and 0.8 m GuHCl represent population-weighted averages of multiple states with marginal stability (Fig. 9*d*) and progressive loss of nonlocal interactions, typical of structures with one-state downhill folding behavior and intrinsically disordered proteins (48).

The work provides new methods for studying p53C aggregation and insights into the physical properties of pre-amyloidogenic states, which may expand the strategies available for preventing p53 aggregation. The ability to trap aggregation precursor states in solution and tune the energetic landscape of p53 affords a unique approach for screening the aggregation propensity of p53 mutants and for testing the effectiveness of treatments that aim to stabilize the native form of p53.

## Experimental procedures

### Protein preparation

The vectors used in this work were prepared and expressed as described previously (29). All chemicals used for protein preparation and fluorescence measurements were of ultrapure grade. For each experiment, proteins were thawed on wet ice and centrifuged at 15,000 × *g* for 10 min at 4 °C, and then protein concentrations were measured based on the absorbance at 280 nm using extinction coefficients of 17,420, 15,930, and 17,285 m^−1^ cm^−1^ for p53C, p63C, and p73C, respectively. Soluble fractions obtained after cell lysis in 50 mm Tris-HCl, pH 7.4, containing 150 mm NaCl, 5 mm DTT, and centrifugation at 18,000 × *g* for 15 min at 4 °C were loaded onto nickel-nitrilotriacetic acid resin (Qiagen) followed by gel filtration chromatography using a Superdex 75 Prepgrade column (GE Life Sciences, catalog no. 17-5174-01). Protein elution was monitored using an Äkta prime system at 280 nm, and purity was checked by 15% SDS-PAGE. The purified proteins were concentrated as required using Amicon Ultra-15 10K filter devices (Millipore, catalog no. UFC901024). p63C and p73C were stored at −80 °C, and 5% glycerol was added to p53C and p53C QM before storage in liquid nitrogen.

### Fluorescence spectroscopy

All experiments were performed using proteins at 5 μm in 50 mm Tris-HCl, pH 7.4, 150 mm NaCl, and 5 mm fresh DTT. For the experiments involving p53C, the samples contained 0.5–1% glycerol. Fluorescence emission measurements were acquired using an ISSK2 spectrofluorometer (ISS Inc.) equipped with a high-pressure cell (ISS Inc.). Samples were excited at 280 nm to measure Tyr + Trp and at 295 nm to measure p53C Trp, and emission was recorded from 290–400 nm. HHP cycles were performed with pressure increments of ∼600 bar, followed by a 5-min interval of pressure stabilization before fluorescence acquisition. Upon excitation at 295 nm, Tyr filtering was performed using a Corning colorless glass filter (WG-280). For light scattering (LS) measurements, the samples were excited at 320 nm, emission was recorded from 300 to 340 nm, and the data were expressed as the area under the LS curve. Changes in the fluorescence spectra for each experimental condition were quantified as the center of spectral mass (*v*) (Equation 1) and then converted to fraction unfolded (α) (Equation 2),

(Eq. 1) $$v=\frac{\sum \lambda F_{i}}{\sum F_{i}}$$

(Eq. 2) $$\alpha =\frac{col\left(v\right)-v_{i}}{v_{f}-v_{i}}$$

where *F_i_* represents the emitted fluorescence at wave number λ. The fraction unfolded was then calculated for each experimental condition (*col*(*v*)) subtracted from its initial value (*v_i_*), and divided by the difference between the last (*v_f_*) and the first value.

### Thermodynamic parameters

Δ*G*_0 M_^F-U^ and Δ*V* values were obtained using the two-state equilibrium assumption of folding/unfolding.

$$\begin{array}{c} F\overset{K_{\text{eq}}}{\to }U\\ \text{Reaction}\;1 \end{array}$$

(Eq. 3) $$K_{\text{eq}}=\frac{\left[U\right]}{\left[F\right]}$$

Considering [*U*] + [*F*] = 1, the fraction of unfolding (α), can be expressed as follows.

(Eq. 4) $$K_{\text{eq}}=\frac{\left[U\right]}{\left[1-U\right]}\approx \frac{\alpha }{1-\alpha }$$

The free energy of denaturation extrapolated at 0 m GuHCl was calculated by the following,

(Eq. 5) $$\Delta G=-RT\;\ln\;K_{\text{eq}}\approx -RT\ln\left(\frac{\alpha }{1-\alpha }\right)$$

where ln(α/(1 − α)) was obtained from the intercept of the linear regression of the transition points. *R* is the universal gas constant (0.00198 kcal·K^−1^·mol^−1^), and *T* is temperature (298 K). If the free energy of unfolding is assumed to evolve linearly with the pressure *p*, then *K*_eq_ varies with Δ*V* as follows.

(Eq. 6) $$\text{d}p\Delta V=-RT\text{d}\;\ln\;K_{\text{eq}}\approx \text{d}\ln\;K_{\text{eq}}=\frac{\Delta V\text{d}p}{-RT}$$

(Eq. 7) $$\;\ln\left(\frac{\alpha }{1-\alpha }\right)=\left(\frac{\Delta V}{RT}\right)p+\ln\;K_{\text{eq}}$$

The Δ*V* values were then calculated from the slope of ln(α/(1 − α)) as a function of *p*, multiplied by *RT* (0.082 liters·atm·K^−1^·mol^−1^ and 298 K, respectively).

### Phasor plots

Experimental data sets consisting of emission fluorescence spectra at increasing concentrations of GuHCl, pressure, or a combination of both were used to calculate a phasor scatter plot, the first harmonic Fourier transformation of the spectral axis *x* (wavelength) and *y* (intensity) using Equations 8 and 9,

(Eq. 8) $$x\;\text{axis}=G=\frac{\sum_{\lambda }I\left(\lambda \right)\cos\left(2\pi n/L\right)}{\sum_{\lambda }I\left(\lambda \right)}$$

(Eq. 9) $$y\;\text{axis}=S=\frac{\sum_{\lambda }I\left(\lambda \right)\sin\left(2\pi n/L\right)}{\sum_{\lambda }I\left(\lambda \right)}$$

where *I* is the fluorescence intensity at each wavelength λ, *n* is the number of the harmonic, and *L* is the wavelength interval recorded. The evaluation of data sets in Fourier space gives the advantage of skipping the two-model assumption for protein folding, F ↔ U, revealing contributions of hidden states in a model-independent manner (33, 34). Phasor plots were calculated using the SimFCS suite.

### Transmission EM

Images were obtained for p53C after incubation of samples for 2 h at 37 °C or under 2.68 kbar at 25 °C. Samples (4 μl) were applied to previously discharged carbon films on 200-mesh copper grids (EMS, catalog no. CF200-cu) for 1 min, gently dried with filter paper, and stained with 2% uranyl acetate for 5 s. Negatively stained images were acquired on a Philips Tecnai microscope operated at 80 kV at ×21,000 and ×46,000 magnification.

### Size-exclusion chromatography

Analytic size exclusion was performed on an ultrafast LC (Shimadzu) system equipped with a Superdex 75 10/300 GL column (GE Life Sciences, catalog no. 17-5174-01). All runs were performed in 50 mm Tris-HCl, pH 7.4, containing 150 mm NaCl, 5 mm fresh DTT, and GuHCl at the specified concentration using a flow of 0.7 ml·min^−1^. Fluorescence emission was monitored at 305 nm upon excitation at 280 nm. p53C (12.5 μm) was incubated in 0.5 or 0.8 m GuHCl for 1 h at 4 °C before injection. Column calibration was performed using thyroglobulin (670 kDa), γ-globulin (158 kDa), ovalbumin (44 kDa), myoglobin (17 kDa), and vitamin B12 (1.35 kDa) (Bio-Rad, catalog no. 151-1901).

### CD spectropolarimetry

CD was carried out on a JASCO spectropolarimeter (J-715). Far-UV spectra were recorded from 260 to 190 nm at 25 °C using 2-nm steps. Mean residue ellipticities [ϴ]_MRE_ in degrees·cm^2^·dmol^−1^ were calculated using the equation, [ϴ] = ϴ/*l*·*c*·10·*n*, where ϴ is the measured ellipticity in millidegrees, *n* is the number of peptide bonds in the primary sequence, *l* is the path length in cm, and *c* is the molar concentration. A circular quartz cell of 0.01-cm path length was used with protein samples of 88.5 μm in 50 mm Tris-HCl, pH 7.4, 150 mm NaCl, and 5 mm DTT. The results are shown as the average ± S.E. of three independent experiments after subtracting the buffer contribution.

### NMR

Heteronuclear ^1^H-^15^N HSQC NMR spectra were acquired at 15 °C in a Bruker Avance III 900-MHz spectrometer equipped with a 5-mm TXI probe (Jiri Jonas National Center of Nuclear Magnetic Resonance, Rio de Janeiro, Brazil). The spectra were acquired with 1,024 points for the ^1^H dimension and 320 points for the ^15^N dimension, with 40 scans (p53C without GuHCl) and 184 scans (p53C in the presence of 0.8, 1.2, and 2 m GuHCl) at each increment. Labeled protein was purified as described before, and concentration was 170 μm in 50 mm Tris-HCl, pH 7.4, containing 150 mm NaCl, 5 mm DTT, and 10% D_2_O. All spectra were processed using TopSpin version 3.2.

### Thioflavin T kinetics

ThT (25 μm) kinetics were recorded using 5 μm p53C in an ISSK2 spectrofluorometer (ISS, Inc.) upon excitation at 450 nm and emission at 477 nm under mild agitation. The reactions were performed in a 1-ml volume in a square cell equipped with magnetic stirrers (Hellma, catalog no. 109004F-10-40), and temperature was controlled (Quantum Northwest, catalog no. TC125) at 37 °C throughout the assay. The experiments were performed at least three times with different protein preparations, and the results are expressed as averages. The protein samples were incubated in 0.5 or 0.8 m GuHCl for 1 h at 4 °C before undergoing direct dilution at 37 °C in the same prewarmed buffer containing the specified GuHCl concentration. Mathematica (Wolfram) was used to calculate *k*_1_ and *k*_2_ values from the fitted curves, as below.

$$\begin{array}{c} N\overset{k_{1}}{\to }I\overset{k_{2}}{\to }U\\ \text{Reaction}\;2 \end{array}$$

*N* and *U* represent the native folded and unfolded states, respectively, and *I* represents the pre-amyloidogenic states.

(Eq. 10) F(t)=m(k1+k2+k2e−k1t−k1e−k2t)(K1+k2)+k3t+a

*F* is the intensity of ThT fluorescence as a function of time (*t*) and amplitude (*m*), defined as the concentration of the monomeric protein multiplied by the raw fluorescence signal of ThT bound to the aggregate at the end of the kinetic experiment. The term *k*_3_*t* is a small linear term related mainly to machine drift, and the parameter *a* is included to allow for a nonzero intercept at *t* = 0.

### Fitting data to the model

The direct application of the law of mass action is generally valid for each step of the reaction, and there is a correspondence between orders and the stoichiometric coefficients. The concentrations at any time, *t*, and the rate laws are given by the following.

(Eq. 11) $$\left(\text{d}\left[N\right]/\text{d}t\right)=-k_{1}\left[N\right]$$

(Eq. 12) $$\left(\text{d}\left[I\right]/\text{d}t\right)=-k_{1}\left[N\right]-k_{2}\left[I\right]$$

(Eq. 13) $$\left(\text{d}\left[U\right]/\text{d}t\right)=k_{2}\left[I\right]$$

Initially, only the native folded form *N* is considered to be present, then at boundary conditions: [*N*]_0_ = [*N*], [*I*]_0_ = 0, [*U*]_0_ = 0, and [*N*]_0_ = [*N*] + [*I*] + [*U*]. Then *N* is obtained from the integration of Equation 11 by the method of separation of variables.

(Eq. 14) $$\int_{{\left[N\right]}_{0}}^{\left[N\right]}\frac{\text{d}\left[N\right]}{\left[N\right]}=\int_{t_{0}}^{t}-k_{1}dt$$

Because *t*_0_ = 0,

(Eq. 15) $$\ln\left[N\right]-\ln{\left[N\right]}_{0}=-k_{1}t$$

(Eq. 16) $$\ln\left(\frac{\left[N\right]}{{\left[N\right]}_{0}}\right)=-k_{1}t$$

(Eq. 17) $$\frac{\left[N\right]}{{\left[N\right]}_{0}}=e^{-k_{1}t}$$

Therefore, the concentration of intermediate, *N*, as function of *t* is given by the following.

(Eq. 18) $$\left[N\right]={\left[N\right]}_{0}e^{-k_{1}t}$$

From Equation 12, the net rate of reaction for *I* depends on the concentrations of both *N* and *I*, where the instantaneous value of [*N*] as a function of reaction time is obtained from Equation 18. Substituting Equation 18 into Equation 12 yields the following.

(Eq. 19) $$\frac{\text{d}\left[I\right]}{\text{d}t}=-k_{1}{\left[N\right]}_{0}e^{-k_{1}t}-k_{2}\left[I\right]$$

This differential equation has a nonexact form, requiring an integrating factor for solution. Considering the following,

(Eq. 20) $$y\prime \left(t\right)=\frac{\text{d}y}{\text{d}t}=q\left(t\right)-py\left(t\right)$$

where *y*, *q*, and *p* are given continuous functions defined as follows,

(Eq. 21) y=[I]

(Eq. 22) $$q\left(t\right)=k_{1}N_{0}e^{-k_{1}t}$$

(Eq. 23) $$p=k_{2}$$

then μ(*t*) would be the following,

(Eq. 24) $$\mu \left(t\right)=e^{\int p\left(t\right)\text{d}t}=e^{pt}$$

and an integrating factor, such that multiplying by *y*′(*t*) (Equation 20) yields Equation 25.

(Eq. 25) μ(t)(y′+py(t))=μ(t)q(t)

According to the product rule of derivatives, the primitive function is as follows,

(Eq. 26) $$\mu \left(t\right)\left(y\prime +py\left(t\right)\right)=\frac{d\left(t\right)\mu \left(t\right)}{\text{d}t}$$

giving the following.

(Eq. 27) $$\frac{d\left(y\left(t\right)\mu \left(t\right)\right)}{\text{d}t}=\mu \left(t\right)q\left(t\right)$$

Integrating both sides with respect to *t*,

(Eq. 28) $$y\left(t\right)=\frac{1}{\mu \left(t\right)}\int \mu \left(t\right)q\left(t\right)\text{d}t$$

and substituting the expression of μ(*t*) defined in Equation 24,

(Eq. 29) $$y\left(t\right)=e^{-pt}\int e^{pt}q\left(t\right)\text{d}t$$

and acquiring the initial consideration of the method, substituting Equations 21–23 and 24 into Equation 28 gives the following.

(Eq. 30) $$\left[I\right]=e^{-k_{2}t}\int e^{k_{2}t}k_{1}{\left[N\right]}_{0}e^{-k_{1}t}\text{d}t$$

The terms *k*_1_ and [*N*]0 are constants, because an exact differential equation is obtained.

(Eq. 31) $$\left[I\right]=e^{-k_{2}t}k_{1}{\left[N\right]}_{0}\int e^{\left(k_{2}-k_{1}\right)t}\text{d}t$$

To solve it, the substitution *u* = (*k*_2_ − *k*_1_)*t* yields (d*u*/d*t*) = (*k*_2_ − *k*_1_); hence, d*t* = (d*u*/(*k*_2_ − *k*_1_). Rewriting Equation 31, defining an interval of integration [0,*u*] and solving yields the following.

(Eq. 32) $$\left[I\right]=e^{-k_{2}t}k_{1}{\left[N\right]}_{0}\frac{1}{\left(K_{2}-k_{1}\right)}\int_{0}^{u}e^{u}du$$

(Eq. 33) $$\left[I\right]=e^{-k_{2}t}k_{1}{\left[N\right]}_{0}\frac{1}{\left(K_{2}-k_{1}\right)}\left(e^{u}-e^{0}+C\right)$$

*C* is an arbitrary constant of integration. Reverting the substitutions of the variable *u* yields the following.

(Eq. 34) $$\left[I\right]=e^{-k_{2}t}k_{1}{\left[N\right]}_{0}\frac{1}{\left(K_{2}-k_{1}\right)}\left(e^{\left(k_{2}-k_{1}\right)t}-1+C\right)$$

Because [*I*]0 = *I*(0) = 0, it defines *C* 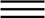 0; therefore, the concentration of *I* at time *t* is as follows.

(Eq. 35) $$\left[I\right]=\frac{{\left[N\right]}_{0}k_{1}}{\left(k_{1}-k_{2}\right)}\left(e^{-k_{2}t}-e^{-k_{1}t}\right)$$

For the final product *U*, the concentrations [*N*], defined by Equation 18, and [*I*], from Equation 35, are substituted in the boundary condition,

(Eq. 36) $${\left[N\right]}_{0}=\left[N\right]+\left[I\right]+\left[U\right]$$

and therefore

(Eq. 37) $$\left[U\right]={\left[N\right]}_{0}-\left[N\right]-\left[I\right]$$

where

(Eq. 38) $$\left[U\right]={\left[N\right]}_{0}-{\left[N\right]}_{0}e^{-k_{1}t}-\frac{{\left[N\right]}_{0}k_{1}}{\left(k_{1}-k_{2}\right)}\left(e^{-k_{2}t}-e^{-k_{1}t}\right)$$

(Eq. 39) $$\left[U\right]={\left[N\right]}_{0}\left(1-e^{-k_{1}t}-\frac{k_{1}}{\left(k_{1}-k_{2}\right)}\left(e^{-k_{2}t}-e^{-k_{1}t}\right)\right)$$

(Eq. 40) $$\left[U\right]={\left[N\right]}_{0}\left(\frac{k_{1}-k_{2}-k_{1}e^{-k_{1}t}+k_{2}e^{-k_{1}t}-k_{1}e^{-k_{2}t}+k_{1}e^{-k_{1}t}}{\left(k_{1}-k_{2}\right)}\right)$$

(Eq. 41) $$\left[U\right]=\frac{{\left[N\right]}_{0}}{k_{1}-k_{2}}\left(k_{1}-k_{2}+k_{2}e^{-k_{1}t}-k_{1}e^{-k_{2}t}\right)$$

Introducing the amplitude, *m* = [*N*]_0_*f*, and the linear terms *k*_3_*t* and *a* for this equation, the concentration of product *U* is associated with the measure of fluorescence emission as a function of time *t* (*F*(*t*)), and is given as follows.

(Eq. 42) $$F\left(t\right)=m\frac{\left(k_{1}-k_{2}+k_{2}e^{-k_{1}t}-k_{1}e^{-k_{2}t}\right)}{\left(k_{1}-k_{2}\right)+k_{3}t}+a$$

## Author contributions

M. M. P., G. A. P. d. O., and J. L. S. designed the research; G. A. P. d. O. and J. L. S. conceived and coordinated the research. M. M. P., A. L. F., M. F. M., M. d. A. M., I. N. S., and G. A. P. d. O. performed the research; J. L. S. and A. M. O. G. contributed new reagents/analytic tools; M. M. P., E. A. C., D. R. N., J. L. S., and G. A. P. d. O. analyzed the data; M. M. P. and G. A. P. d. O. prepared figures; E. G. wrote the program for the spectral phasor analysis; A. I. performed the NMR experiments. The manuscript was prepared by M. M. P. and G. A. P. d. O. with edits by E. A. C., E. G., and J. L. S.

## Footnotes

5 The abbreviations used are:

MG: molten globule
GuHCl: guanidine hydrochloride
HHP: high hydrostatic pressure
bis-ANS: 4,4′-dianilino-1,1′-binaphthyl-5,5′-disulfonic acid
HSQC: heteronuclear single quantum coherence
QM: quadruple mutant
MD: molecular dynamics
ThT: thioflavin T
BHB: backbone hydrogen bonds
LS: light scattering.

## References

[1] Wolf D., Harris N., and Rotter V. (1984) Reconstitution of p53 expression in a nonproducer Ab-MuLV-transformed cell line by transfection of a functional p53 gene. Cell 38, 119–126 10.1016/0092-8674(84)90532-4 6088057

[2] Milner J., and Medcalf E. A. (1991) Cotranslation of activated mutant p53 with wild type drives the wild-type p53 protein into the mutant conformation. Cell 65, 765–774 10.1016/0092-8674(91)90384-B 2040013

[3] Halevy O., Michalovitz D., and Oren M. (1990) Different tumor-derived p53 mutants exhibit distinct biological activities. Science 250, 113–116 10.1126/science.2218501 2218501

[4] Costa D. C., de Oliveira G. A., Cino E. A., Soares I. N., Rangel L. P., and Silva J. L. (2016) Aggregation and prion-like properties of misfolded tumor suppressors: is cancer a prion disease? Cold Spring Harb. Perspect. Biol. 8, a023614 10.1101/cshperspect.a023614 27549118 PMC5046694

[5] Ano Bom A. P., Rangel L. P., Costa D. C., de Oliveira G. A., Sanches D., Braga C. A., Gava L. M., Ramos C. H., Cepeda A. O., Stumbo A. C., De Moura Gallo C. V., Cordeiro Y., and Silva J. L. (2012) Mutant p53 aggregates into prion-like amyloid oligomers and fibrils: implications for cancer. J. Biol. Chem. 287, 28152–28162 10.1074/jbc.M112.340638 22715097 PMC3431633

[6] Levy C. B., Stumbo A. C., Ano Bom A. P., Portari E. A., Carneiro Y., Silva J. L., and De Moura-Gallo C. V. (2011) Co-localization of mutant p53 and amyloid-like protein aggregates in breast tumors. Int. J. Biochem. Cell Biol. 43, 60–64 10.1016/j.biocel.2010.10.017 21056685

[7] Yang-Hartwich Y., Soteras M. G., Lin Z. P., Holmberg J., Sumi N., Craveiro V., Liang M., Romanoff E., Bingham J., Garofalo F., Alvero A., and Mor G. (2015) p53 protein aggregation promotes platinum resistance in ovarian cancer. Oncogene 34, 3605–3616 10.1038/onc.2014.296 25263447

[8] Ghosh S., Salot S., Sengupta S., Navalkar A., Ghosh D., Jacob R., Das S., Kumar R., Jha N. N., Sahay S., Mehra S., Mohite G. M., Ghosh S. K., Kombrabail M., Krishnamoorthy G., Chaudhari P., and Maji S. K. (2017) p53 amyloid formation leading to its loss of function: implications in cancer pathogenesis. Cell Death Differ. 24, 1784–1798 10.1038/cdd.2017.105 28644435 PMC5596421

[9] De Smet F., Saiz Rubio M., Hompes D., Naus E., De Baets G., Langenberg T., Hipp M. S., Houben B., Claes F., Charbonneau S., Delgado Blanco J., Plaisance S., Ramkissoon S., Ramkissoon L., Simons C., et al (2017) Nuclear inclusion bodies of mutant and wild-type p53 in cancer: a hallmark of p53 inactivation and proteostasis remodelling by p53 aggregation. J. Pathol. 242, 24–38 10.1002/path.4872 28035683

[10] Silva J. L., Cino E. A., Soares I. N., Ferreira V. F., and de Oliveira G. A. P. (2018) Targeting the prion-like aggregation of mutant p53 to combat cancer. Acc. Chem. Res. 51, 181–190 10.1021/acs.accounts.7b00473 29260852

[11] Friedler A., DeDecker B. S., Freund S. M., Blair C., Rüdiger S., and Fersht A. R. (2004) Structural distortion of p53 by the mutation R249S and its rescue by a designed peptide: implications for “mutant conformation”. J. Mol. Biol. 336, 187–196 10.1016/j.jmb.2003.12.005 14741214

[12] Issaeva N., Friedler A., Bozko P., Wiman K. G., Fersht A. R., and Selivanova G. (2003) Rescue of mutants of the tumor suppressor p53 in cancer cells by a designed peptide. Proc. Natl. Acad. Sci. U.S.A. 100, 13303–13307 10.1073/pnas.1835733100 14595027 PMC263793

[13] Friedler A., Hansson L. O., Veprintsev D. B., Freund S. M., Rippin T. M., Nikolova P. V., Proctor M. R., Rüdiger S., and Fersht A. R. (2002) A peptide that binds and stabilizes p53 core domain: chaperone strategy for rescue of oncogenic mutants. Proc. Natl. Acad. Sci. U.S.A. 99, 937–942 10.1073/pnas.241629998 11782540 PMC117409

[14] Soragni A., Janzen D. M., Johnson L. M., Lindgren A. G., Thai-Quynh Nguyen A., Tiourin E., Soriaga A. B., Lu J., Jiang L., Faull K. F., Pellegrini M., Memarzadeh S., and Eisenberg D. S. (2016) A designed inhibitor of p53 aggregation rescues p53 tumor suppression in ovarian carcinomas. Cancer Cell 29, 90–103 10.1016/j.ccell.2015.12.002 26748848 PMC4733364

[15] Ishimaru D., Ano Bom A. P., Lima L. M., Quesado P. A., Oyama M. F., de Moura Gallo C. V., Cordeiro Y., and Silva J. L. (2009) Cognate DNA stabilizes the tumor suppressor p53 and prevents misfolding and aggregation. Biochemistry 48, 6126–6135 10.1021/bi9003028 19505151

[16] Kovachev P. S., Banerjee D., Rangel L. P., Eriksson J., Pedrote M. M., Martins-Dinis M. M. D. C., Edwards K., Cordeiro Y., Silva J. L., and Sanyal S. (2017) Distinct modulatory role of RNA in the aggregation of the tumor suppressor protein p53 core domain. J. Biol. Chem. 292, 9345–9357 10.1074/jbc.M116.762096 28420731 PMC5454114

[17] Joerger A. C., Bauer M. R., Wilcken R., Baud M. G. J., Harbrecht H., Exner T. E., Boeckler F. M., Spencer J., and Fersht A. R. (2015) Exploiting transient protein states for the design of small-molecule stabilizers of mutant p53. Structure 23, 2246–2255 10.1016/j.str.2015.10.016 26636255 PMC4671956

[18] Basse N., Kaar J. L., Settanni G., Joerger A. C., Rutherford T. J., and Fersht A. R. (2010) Toward the rational design of p53-stabilizing drugs: probing the surface of the oncogenic Y220C mutant. Chem. Biol. 17, 46–56 10.1016/j.chembiol.2009.12.011 20142040

[19] Baldwin R. L., and Rose G. D. (2013) Molten globules, entropy-driven conformational change and protein folding. Curr. Opin. Struct. Biol. 23, 4–10 10.1016/j.sbi.2012.11.004 23237704

[20] de Oliveira G. A. P., and Silva J. L. (2017) The push-and-pull hypothesis in protein unfolding, misfolding and aggregation. Biophys. Chem. 231, 20–26 10.1016/j.bpc.2017.03.007 28377183

[21] Christensen H., and Pain R. H. (1991) Molten globule intermediates and protein folding. Eur. Biophys. J. 19, 221–229 2060495 10.1007/BF00183530

[22] Yang J., Dunker A. K., Powers J. R., Clark S., and Swanson B. G. (2001) β-lactoglobulin molten globule induced by high pressure. J. Agric. Food Chem. 49, 3236–3243 10.1021/jf001226o 11453757

[23] Jha S. K., and Marqusee S. (2014) Kinetic evidence for a two-stage mechanism of protein denaturation by guanidinium chloride. Proc. Natl. Acad. Sci. U.S.A. 111, 4856–4861 10.1073/pnas.1315453111 24639503 PMC3977270

[24] Gerber R., Tahiri-Alaoui A., Hore P. J., and James W. (2007) Oligomerization of the human prion protein proceeds via a molten globule intermediate. J. Biol. Chem. 282, 6300–6307 10.1074/jbc.M608926200 17210575

[25] Skora L., Becker S., and Zweckstetter M. (2010) Molten globule precursor states are conformationally correlated to amyloid fibrils of human β-2-microglobulin. J. Am. Chem. Soc. 132, 9223–9225 10.1021/ja100453e 20565073

[26] Karamanos T. K., Pashley C. L., Kalverda A. P., Thompson G. S., Mayzel M., Orekhov V. Y., and Radford S. E. (2016) A population shift between sparsely populated folding intermediates determines amyloidogenicity. J. Am. Chem. Soc. 138, 6271–6280 10.1021/jacs.6b02464 27117876 PMC4922733

[27] Ceru S., and Zerovnik E. (2008) Similar toxicity of the oligomeric molten globule state and the prefibrillar oligomers. FEBS Lett. 582, 203–209 10.1016/j.febslet.2007.12.002 18078819

[28] Bom A. P., Freitas M. S., Moreira F. S., Ferraz D., Sanches D., Gomes A. M., Valente A. P., Cordeiro Y., and Silva J. L. (2010) The p53 core domain is a molten globule at low pH: functional implications of a partially unfolded structure. J. Biol. Chem. 285, 2857–2866 10.1074/jbc.M109.075861 19933157 PMC2807339

[29] Cino E. A., Soares I. N., Pedrote M. M., de Oliveira G. A., and Silva J. L. (2016) Aggregation tendencies in the p53 family are modulated by backbone hydrogen bonds. Sci. Rep. 6, 32535 10.1038/srep32535 27600721 PMC5013286

[30] Wang G., and Fersht A. R. (2017) Multisite aggregation of p53 and implications for drug rescue. Proc. Natl. Acad. Sci. U.S.A. 114, E2634–E2643 10.1073/pnas.1700308114 28292898 PMC5380089

[31] Wang G., and Fersht A. R. (2015) Mechanism of initiation of aggregation of p53 revealed by Φ-value analysis. Proc. Natl. Acad. Sci. U.S.A. 112, 2437–2442 10.1073/pnas.1500243112 25675526 PMC4345617

[32] Joerger A. C., Allen M. D., and Fersht A. R. (2004) Crystal structure of a superstable mutant of human p53 core domain: insights into the mechanism of rescuing oncogenic mutations. J. Biol. Chem. 279, 1291–1296 10.1074/jbc.M309732200 14534297

[33] James N. G., Ross J. A., Stefl M., and Jameson D. M. (2011) Applications of phasor plots to *in vitro* protein studies. Anal. Biochem. 410, 70–76 10.1016/j.ab.2010.11.011 21078289 PMC3021620

[34] Malacrida L., Gratton E., and Jameson D. M. (2015) Model-free methods to study membrane environmental probes: a comparison of the spectral phasor and generalized polarization approaches. Methods Appl. Fluoresc. 3, 047001 10.1088/2050-6120/3/4/047001 27182438 PMC4862737

[35] Silva J. L., Oliveira A. C., Vieira T. C., de Oliveira G. A., Suarez M. C., and Foguel D. (2014) High-pressure chemical biology and biotechnology. Chem. Rev. 114, 7239–7267 10.1021/cr400204z 24884274

[36] Royer C. A. (2006) Probing protein folding and conformational transitions with fluorescence. Chem. Rev. 106, 1769–1784 10.1021/cr0404390 16683754

[37] Das A., and Mukhopadhyay C. (2009) Urea-mediated protein denaturation: a consensus view. J. Phys. Chem. B 113, 12816–12824 10.1021/jp906350s 19708649

[38] Zhou R., Li J., Hua L., Yang Z., and Berne B. J. (2011) Comment on “urea-mediated protein denaturation: a consensus view”. J. Phys. Chem. B 115, 1323–1326; discussion 1327–1328 10.1021/jp105160a 21247088 PMC3050030

[39] Roche J., Caro J. A., Norberto D. R., Barthe P., Roumestand C., Schlessman J. L., García A. E., Garcia-Moreno B. E., and Royer C. A. (2012) Cavities determine the pressure unfolding of proteins. Proc. Natl. Acad. Sci. U.S.A. 109, 6945–6950 10.1073/pnas.1200915109 22496593 PMC3344970

[40] de Oliveira G. A., and Silva J. L. (2015) A hypothesis to reconcile the physical and chemical unfolding of proteins. Proc. Natl. Acad. Sci. U.S.A. 112, E2775–E2784 10.1073/pnas.1500352112 25964355 PMC4450381

[41] Borgohain G., and Paul S. (2018) The opposing effect of urea and high pressure on the conformation of the protein β-hairpin: a molecular dynamics simulation study. J. Mol. Liquids 251, 378–384 10.1016/j.molliq.2017.12.054

[42] Baldwin R. L., Frieden C., and Rose G. D. (2010) Dry molten globule intermediates and the mechanism of protein unfolding. Proteins 78, 2725–2737 10.1002/prot.22803 20635344 PMC2927783

[43] Jha S. K., and Udgaonkar J. B. (2009) Direct evidence for a dry molten globule intermediate during the unfolding of a small protein. Proc. Natl. Acad. Sci. U.S.A. 106, 12289–12294 10.1073/pnas.0905744106 19617531 PMC2718347

[44] Reiner A., Henklein P., and Kiefhaber T. (2010) An unlocking/relocking barrier in conformational fluctuations of villin headpiece subdomain. Proc. Natl. Acad. Sci. U.S.A. 107, 4955–4960 10.1073/pnas.0910001107 20194774 PMC2841885

[45] Dasgupta A., Udgaonkar J. B., and Das P. (2014) Multistage unfolding of an SH3 domain: an initial urea-filled dry molten globule precedes a wet molten globule with non-native structure. J. Phys. Chem. B 118, 6380–6392 10.1021/jp410019f 24661021

[46] Acharya N., Mishra P., and Jha S. K. (2016) Evidence for dry molten globule-like domains in the pH-induced equilibrium folding intermediate of a multidomain protein. J. Phys. Chem. Lett. 7, 173–179 10.1021/acs.jpclett.5b02545 26700266

[47] Neumaier S., and Kiefhaber T. (2014) Redefining the dry molten globule state of proteins. J. Mol. Biol. 426, 2520–2528 10.1016/j.jmb.2014.04.022 24792909

[48] Muñoz V., Campos L. A., and Sadqi M. (2016) Limited cooperativity in protein folding. Curr. Opin. Struct. Biol. 36, 58–66 10.1016/j.sbi.2015.12.001 26845039

[49] Sadqi M., Fushman D., and Muñoz V. (2006) Atom-by-atom analysis of global downhill protein folding. Nature 442, 317–321 10.1038/nature04859 16799571

[50] Cho S. S., Weinkam P., and Wolynes P. G. (2008) Origins of barriers and barrierless folding in BBL. Proc. Natl. Acad. Sci. U.S.A. 105, 118–123 10.1073/pnas.0709376104 18172203 PMC2224170

[51] Cañadillas J. M., Tidow H., Freund S. M., Rutherford T. J., Ang H. C., and Fersht A. R. (2006) Solution structure of p53 core domain: structural basis for its instability. Proc. Natl. Acad. Sci. U.S.A. 103, 2109–2114 10.1073/pnas.0510941103 16461916 PMC1413739

[52] Enthart A., Klein C., Dehner A., Coles M., Gemmecker G., Kessler H., and Hagn F. (2016) Solution structure and binding specificity of the p63 DNA binding domain. Sci. Rep. 6, 26707 10.1038/srep26707 27225672 PMC4880913

[53] Canning P., von Delft F., and Bullock A. N. (2012) Structural basis for ASPP2 recognition by the tumor suppressor p73. J. Mol. Biol. 423, 515–527 10.1016/j.jmb.2012.08.005 22917970 PMC3472557

